# A Dynamic Range Enhanced Readout Technique with a Two-Step TDC for High Speed Linear CMOS Image Sensors

**DOI:** 10.3390/s151128224

**Published:** 2015-11-06

**Authors:** Zhiyuan Gao, Congjie Yang, Jiangtao Xu, Kaiming Nie

**Affiliations:** School of Electronic Information Engineering, Tianjin University, 92 Weijin Road, Nankai District, Tianjin 300072, China; E-Mails: gaozhiyuan@tju.edu.cn (Z.G.); yangcongjie@tju.edu.cn (C.Y.); nkaiming@tju.edu.cn (K.N.)

**Keywords:** CMOS image sensor, CTIA, wide dynamic range, two-step TDC, error calibration

## Abstract

This paper presents a dynamic range (DR) enhanced readout technique with a two-step time-to-digital converter (TDC) for high speed linear CMOS image sensors. A multi-capacitor and self-regulated capacitive trans-impedance amplifier (CTIA) structure is employed to extend the dynamic range. The gain of the CTIA is auto adjusted by switching different capacitors to the integration node asynchronously according to the output voltage. A column-parallel ADC based on a two-step TDC is utilized to improve the conversion rate. The conversion is divided into coarse phase and fine phase. An error calibration scheme is also proposed to correct quantization errors caused by propagation delay skew within −*T*_clk_~+*T*_clk_. A linear CMOS image sensor pixel array is designed in the 0.13 μm CMOS process to verify this DR-enhanced high speed readout technique. The post simulation results indicate that the dynamic range of readout circuit is 99.02 dB and the ADC achieves 60.22 dB SNDR and 9.71 bit ENOB at a conversion rate of 2 MS/s after calibration, with 14.04 dB and 2.4 bit improvement, compared with SNDR and ENOB of that without calibration.

## 1. Introduction

Linear array image sensors obtain continuous images by scanning the target objects in one direction or in a loop, thus, they are usually used in industry detection, aerial photography, and satellite imaging. More and more applications require high speed scans to improve detection efficiency, and in some cases, the target objects are moving fast. Both conditions increase the demands of high line rate linear image sensors. Moreover, the target scene may contain high light and low light objects, and the dynamic range of the targets usually exceeds 90 dB. However, the dynamic range of traditional readout circuits is limited to 60–70 dB [1,2,3]. A wide dynamic range readout circuit is expected to cover at least a detecting range over 90 dB to capture all the possible information [1,4]. Therefore, the readout circuits of linear array image sensors require a wide detecting range and high speed readout.

For a linear CMOS image sensor (CIS), different types of readout techniques are used for various purposes, mainly including direct injection (DI), gate modulated injection (GMI), source follower per detector (SFD), and capacitor trans-impedance amplifier (CTIA) [5], *etc*. However, the integration linearity of DI and SFD gets significantly worse in dealing with wide range input signals. In GMI, the current gain and injection efficiency are sensitive to the threshold voltage change of MOS transistors, thus, readout circuits of different pixels may have large offset voltage [6]. Among these, CTIA can achieve good integration linearity and high injection efficiency, which makes it a popular readout circuit structure.

In order to fulfill the requirement of 90 dB photon dynamic range, a dynamic range extension technique has to be utilized. Various solutions for wide dynamic range CIS have been reported as CIS technology has developed. Some of them take advantage of improved pixel architectures, while others use novel readout methods. In general, they can be categorized into four main methods, *i.e.*, logarithmic response, well capacity adjustment, multiple sampling, and saturation detection [7,8,9]. Well capacity adjustment and saturation detection are usually used in 4-T pixels. Logarithmic response and multiple sampling methods can be used with CTIA readout circuits. However, multiple sampling needs two or more times the readout operations than traditional pixels, which significantly limits the line rate. Logarithmic response is a simple and effective way, however, it suffers from fixed pattern noise when applied to APS. Therefore, a logarithmic-like response curve is created with a new CTIA-based dynamic range extension technique to achieve over 90 dB photon dynamic range.

High speed ADC is also necessary to achieve high line rates. Column-parallel ADC is the most widely used architecture in high speed linear imagers because it provides a better tradeoff among readout speed, silicon area and power consumption. Currently, three types of column-parallel ADCs have been used in CMOS image sensors: a successive approximation (SAR) ADC [10,11], a cyclic ADC [12], and a single-slope (SS) ADC [13,14]. Compared with SAR and cyclic ADCs, SS ADCs have many attractive features such as good linearity, simplicity of the analog circuits and resulting suitability for fine-pitched pixels. Moreover, they can ensure uniformity between columns and thus minimize column FPN. However, it may be difficult to simultaneously achieve both high-speed A/D conversion and high bit resolution because the required conversion time increases by a factor of 2^N^ for N-bit resolution. To improve the conversion speed, some new methods have been reported: two-step single-slope ADC [15,16,17], multiple-ramp multiple-slope (MRMS) ADC [18], *etc.* In addition, further reduction in conversion time can be achieved by time-based SS ADC [19]. The input signal is converted to a time-domain representation using a ramp generator and a comparator and then the time domain information is quantized by a time-to-digital converter (TDC).

This paper presents a dynamic range enhanced, high speed readout technique consisting of a CTIA type readout circuit and a column-parallel SS ADC with two-step TDC. The proposed ADC has a significantly increased conversion rate compared with the conventional SS ADC [13,14], and costs less power and area than the multiple-ramp ADC [20].

The rest of this paper is organized as follows: Section 2 describes dynamic range enhanced column readout circuits as a preliminary to the following sections. Section 3 discusses the detailed design and operation of proposed ADC, a calibration technique to mitigate the impact of mismatch in the two-step TDC is also presented. Section 4 presents the results, and Section 5 provides the conclusions.

## 2. Dynamic Range Enhanced Readout Circuit

**Figure 1:**
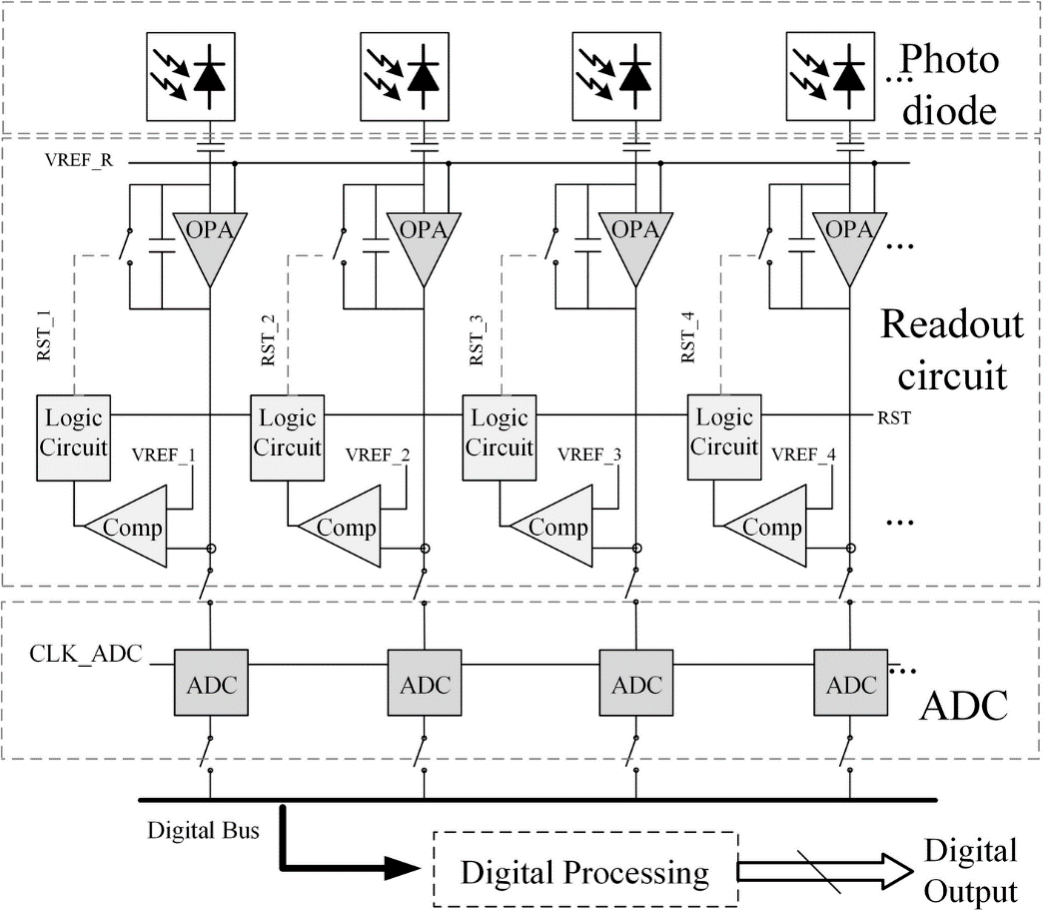
Block diagram of a linear image sensor.

The architecture of a linear image sensor with dynamic range enhanced readout circuits and two-step TDC based ADCs is shown in Figure 1. It consists of a linear photodiode array, dynamic range enhanced readout circuits, analog to digital converters, and peripheral circuits. The photodiode and readout circuit compose a pixel in the conventional sensors.

### 2.1. Capacitor Trans-Impedance Amplifier (CTIA)

An improved CTIA structure is developed as the readout circuit in this paper due to its wide detection range, low noise and good integration linearity. Moreover, the non-destructive readout (NDR) can be realized with the CTIA structure. NDR mode is similar to the standard readout mode except that the output signals can be read out multiple times in a single readout integration time. The CTIA isolates the photodiode and the output signals by an amplifier, thus the readout operation does not have any effect on the photodiode.

**Figure 2:**
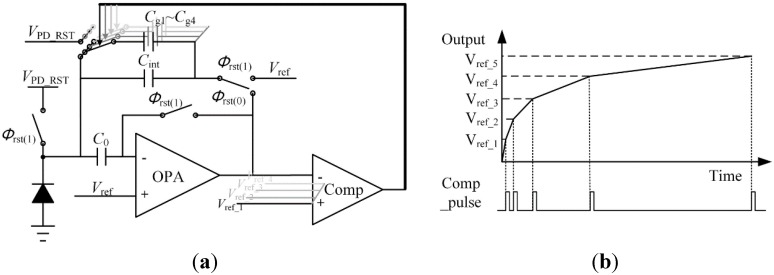
(**a**) The structure of CTIA; (**b**) The output curve of CTIA.

The structure of CTIA is shown in Figure 2, where the integration capacitor is placed on the feedback loop of the amplifier with a reset switch to initialize the integration capacitor and reset the output to *V*_ref_. Additional integration capacitors *C*_g1_ to *C*_g4_ are connected to the circuit in parallel with *C*_int_. The left plates of *C*_g1_ to *C*_g4_ are connected to *V*_PD_RST_ in reset phase. A comparator at the output node is used to judge different reference voltages and control the amount of integrating capacitors accessing to CTIA at integration period. This CTIA-based structure has offset cancellation ability and dynamic range extension function.

#### 2.1.1. Offset Cancellation

Device mismatch and process variations may lead to offsets among pixels. A conventional CTIA-based pixel resets the photodiode by shorting the negative input and output of the OPA [21]. This method is simple, but an offset voltage induced by the OPA exists at the output, which is one of the sources of fixed pattern noise in pixel arrays. In this design, a reset transistor, an extra capacitor *C*_0_ and a Single Pole Double Throw (SPDT) switch are adopted to eliminate the offsets at the output, also make the reset voltage of the photodiode flexible.

**Figure 3:**
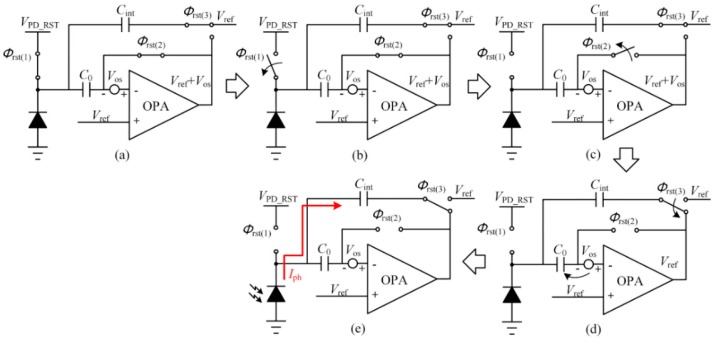
Switch sequence of the offset cancellation scheme. (**a**) Reset state; (**b**) *ϕ*_rst(1)_ is switched off; (**c**) *ϕ*_rst(2)_ is switched off; (**d**) *ϕ*_rst(3)_ is switched from *V*_ref_ to the output terminal; (**e**) *I*_ph_ starts to integrate on *C*_int_.

The switch sequence is shown in Figure 3. During the reset period, *Φ*_rst(1)–(3)_ signals are set as the logic high level to turn these switches on, as shown in Figure 3a. At the reset period, the photodiode is reset to *V*_PD_RST_, and the negative input and the output of the OPA is reset to *V*_ref_ + *V*_os_, where *V*_os_ represents the offset voltage induced by the mismatches in the OPA. At the same time, the right plate of the integrating capacitor is always connected to *V*_ref_.

The reset phase ends as *Φ*_rst(1)_ transits to its low level first as Figure 3b shows. *KT/C* noise is integrated on the photodiode, but it cannot propagate to the output as the right plates of *C*_int_ and *C*_0_ are still clamped. Then *Φ*_rst(2)_ transits to its low level as Figure 3c shows. The offset voltage is stored on *C*_0_ while *V*_out_ remains as *V*_ref_ + *V*_os_. When *Φ*_rst(3)_ is switched to the output of the OPA as shown in Figure 3d, because the charge stored on the integration capacitor *C*_int_ and *C*_0_ remains and they form a negative feedback, *V*_out_ turns from *V*_ref_ + *V*_os_ to *V*_ref_. In fact, the accurate value of *V*_out_ is:

(1) $$V_{\text{out}}\text{=}V_{\text{ref}}-\frac{V_{\text{os}}}{A-1}$$

where *A* is the open-loop gain of the OPA in the CTIA structure. By considering the load effect of the capacitors, the frequency expression of the open-loop gain in a two-stage OPA is:

(2) $$A\text{(}s\text{) = }\frac{A_{0}}{\left(1+R_{\text{I}}C_{\text{I}}s\right)\cdot \left(1+R_{\text{II}}C_{\text{II}}s\right)}$$

where *A*_0_ is the open-loop DC gain, *R*_I_ (*R*_II_) and *C*_I_ (*C*_II_) are the resistance and capacitance to ground seen from the output of the first (second) stage. The capacitance load at output is derived as:

(3) $$\left\{ \begin{array}{l} C_{\text{II}}\left(\text{reset}\text{phase}\right)\text{ = }C_{\text{int}}\\ C_{\text{II}}\left(\text{integration}\text{phase}\right)\text{ = }\frac{\left(\frac{C_{0}\cdot C_{\mathrm{in}}}{C_{0}\text{ + }C_{\mathrm{in}}}\text{ + }C_{\text{pd}}\right)\cdot C_{\text{int}}}{\left(\frac{C_{0}\cdot C_{\mathrm{in}}}{C_{0}\text{ + }C_{\mathrm{in}}}\text{ + }C_{\text{pd}}\right)\text{+}C_{\text{int}}}\approx \frac{C_{0}\cdot C_{\mathrm{in}}}{C_{0}\text{+}C_{\mathrm{in}}}\text{ + }C_{\text{pd}} \end{array}\right.$$

where *C*_in_ is the parasitic capacitance of input terminal, and *C*_pd_ is the parasitic capacitance of the photodiode. Since they are not large capacitance load, the OPA may achieve a good transient response characteristic easily. Furthermore, when the signal settles, the open-loop gain at low frequency is extremely large. Therefore, in Equation (1), the latter term is so small that it should be omitted, *i.e.*, the offset voltage at output terminal is cancelled. Then the CTIA structure starts to integrate the photo current *I*_ph_ on the integration capacitor *C*_int_ from *V*_ref_. Since the OPA is not ideal, its gain and the parasitic capacitance of the photodiode should be taken into consideration. Then the output voltage is derived as:

(4) $$V_{\text{out}}=\frac{A}{1+A}\left(V_{\text{ref}}+\frac{\left(A-1\right)I_{\text{ph}}\cdot t}{\left(A-1\right)C_{\mathrm{int}}+C_{\text{pd}}}\right)$$

where *t* is the integration time. From the equation above, if *A* is large enough, the expression of *V*_out_ can be simplified as:

(5) $$V_{\text{out}}=V_{\text{ref}}+I_{\text{ph}}\cdot t/C_{\mathrm{int}}$$

#### 2.1.2. Dynamic Range Extension

In the conventional integration, the output increases linearly and saturates at *V*_sat_ as Equation (5). CTIA operates as an integration circuit for photodiode. The photocurrent integrates on the capacitors connected across the input and output terminal which determines the sensitivity of integration. Sensitivity here is defined as the voltage variation under the conditions of 1 A photo current and 1 s integration time. Then the sensitivity could be derived from Equation (5) as:

(6) $$S\text{=}\frac{1}{C_{\mathrm{int}}}$$

High sensitivity of integration can be achieved by using a small capacitance, and a large capacitance achieves the opposite [22]. A good sensitivity could improve the ability of low light detection and achieve low equivalent input noise, but it gets saturated under high light conditions. To address this issue, a multi-capacitor and self-regulation method is applied to the CTIA structure to achieve the wide dynamic range feature. Several capacitors, *C*_g1_ to *C*_g4_ in this design, are connected into the circuits in parallel with *C*_int_ as additional integration capacitors. A reusable comparator is connected to the output terminal of the CTIA to monitor the signal voltage. The corresponding reference voltages, *V*_ref1_ to *V*_ref5_ are delivered by a MUX to the input terminal of the comparator. The sensitivity of the CTIA is automatically adjusted by switching different capacitors to the integration node asynchronously according to the output voltage. This method extends the dynamic range by compressing the photo response in the high light region as shown in Figure 2b. With a logarithmic-like photoresponse curve, the output under high light conditions would not get saturated compared to the traditional design with a single integration capacitor. Then both low and high light information is acquired.

The principle of this dynamic range extension approach is detailed as follows. When the reset phase ends, the photocurrent begins to integrate on *C*_int_, and *V*_out_ grows upward from *V*_ref_. During the integration period, the comparator keeps monitoring *V*_out_, and the reference voltage is set as V_ref1_ at first. Once the signal voltage reaches the reference voltage *V*_ref1_, a short pulse is generated to switch *C*_g1_ to the integration node. Thus, the integration capacitance increases and the sensitivity gets lower. After these operations, the reference voltage connected to the comparator is switched to the next predetermined voltage, *V*_ref2_, and then the comparator continues to monitor the signal and repeats the operations above. Assuming that light intensity is constant, then the output voltage of the CTIA is:

(7) $$V_{\text{out}}\text{ = }I_{\text{ph}}\cdot \sum_{\text{i = }1}^{\text{n}}\frac{t_{\text{i}}}{C_{\text{total}\_i}}+V_{\text{ref}}$$

where *t*_i_ is the integration time of *i*-th segment, and C_total_i_ is the total capacitance used for photo current integration.

Thus, the sensitivity of each integration segment is:

(8) $$S_{\text{i}}\text{ = }\frac{1}{C_{\text{total}\_i}}$$

In the proposed design, four additional capacitors, *C*_g1_ to *C*_g4_, are to be connected to the integration node, and five reference voltages (including a saturation voltage) are alternately applied to the comparator. Then the photo response curve becomes a polyline with five segments before it gets saturated. The output swing in this design is from 1 V to 3 V, which is divided into five segments, thus the reference voltages are 1.4, 1.8, 2.2, 2.6 and 3.0 V, respectively.

### 2.2. Readout Circuits Implementation

In this design, a polyline type photoresponse curve with five segments is expected. The slope ratios of two adjacent segments are assumed to be equal. Then the slope ratio *k* can be calculated by:

(9) $$k\text{= 1 + }\frac{C_{\text{n}}}{\sum_{\text{i = 0}}^{\text{n-1}}C_{\text{i}}}(0\leq n\leq 4)$$

where n is the number of capacitors, *C*_n_ is the *n*-th capacitor that connected to the integration node. Each capacitance can be calculated by detection range and *k*. In this design, we define the maximum saturate current *I*_0_ to be around 12 μA within an integration time of 2 μs as the target. The slope ratios of two adjacent segments are assumed to be equal, and the voltage swing of the readout circuit is 2 V. According to these conditions, k is figured out to be 3.21. The initial capacitance is 400 fF, then four additional capacitors *C*_1_–*C*_4_ are 0.88 pF, 2.84 pF, 9.02 pF, and 29.03 pF. The total capacitance is 42.17 pF.

In order to capture clear image in fast scan application, the integration time in this design is as short as 2 μs. The maximum saturation current *I*_0_ during this time is 12.3 μA, then the maximum charging current *I*_total_max_ at the first integration segment is given by:

(10) $$I_{\text{total\_max}}\text{=}I_{0}\times \frac{C_{\text{total}}}{C_{0}}$$

From Equation (10), *I*_total_max_ is around 1.3 mA, *i.e.*, the CTIA has to output 1.3 mA current to drive all these capacitors.

#### 2.2.1. OPA Design

**Figure 4:**
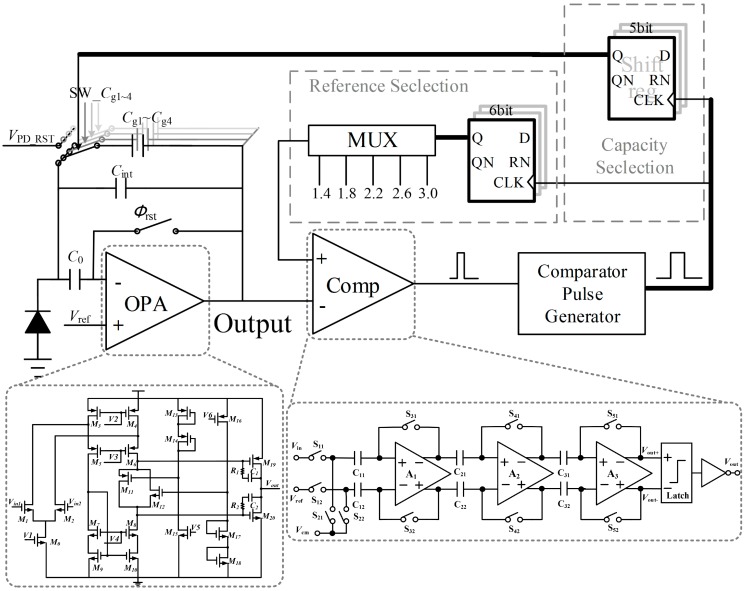
Circuit diagram of CTIA.

The OPA should be carefully designed to obtain such a large output current and reduce quiescent current as well. As shown in Figure 4, a class AB output stage is employed in OPA design. In order to achieve high speed detection, the amplifier may need to supply significant amounts of current to rapidly charge the load to the target accuracy. Therefore, an OPA with high gain, large output current, and low quiescent current is required. In this design, the OPA with class AB output stage achieves 102 dB gain and over 1.3 mA output current with a 130 μA quiescent current.

#### 2.2.2. Logic Control and Comparator Design

As shown in Figure 4, the control logic circuit is mainly composed of a pulse generator (PG), a reference voltage selector (RVS) and an integrating capacitor selector (ICS). PG is used to broaden the width of the reset pulse generated by the comparator when it is triggered. RVS delivers the corresponding reference voltage to the input of the comparator. ICS controls the amount of integrating capacitors accessing to CTIA for integration.

To acquire an accurate integrated voltage, the comparator should have sufficient gain, as well as the smallest propagation delay. The comparator uses three cascaded low-gain amplifiers as the preamplifier and a latch at the output, as shown in Figure 4. An offset cancellation technique is utilized to compensate the offsets of these amplifiers. As a result, the input offset of the comparator can be reduce to less than 0.5LSB.

## 3. Design of Column-Level ADC with Two-Step TDC

The conventional column-level ADC usually operates at a relatively slow conversion speed. To achieve both wide dynamic range and high conversion rate, with low power consumption, a two-step TDC is utilized in this design.

### 3.1. Operation Principle

The basic concept of the proposed ADC consists of an analog-to-time conversion (ATC) and a parallel two-step TDC. The ATC transforms the sampled input voltage into a pulse signal whose width is linearly proportional to the input voltage. Then the width of the pulse signal is measured by the parallel two-step TDC, which yields a corresponding digital output. The n-bit conversion process of TDC is divided into *m*-bit coarse and *l*-bit fine conversions, where *n = m + l.* The coarse quantization is implemented by a counter which consumes little power, and the fine quantization is performed by a Vernier delay line (VDL) to obtain good accuracy [23]. By using this two-step architecture, the power consumption of TDC is reduced while maintaining large quantization range and fine resolution. The key challenges of this architecture are to achieve an efficient two-step TDC circuit architecture and ensuring the synchronization of two-step quantization.

### 3.2. ATC

**Figure 5:**
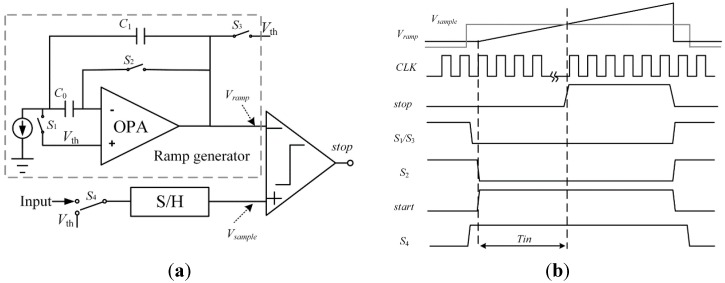
(**a**) Block diagram of ATC; (**b**) Timing diagram of ATC.

Figure 5 shows the block diagrams and timing diagrams of the suggested linear ATC, which is composed of a sample-and-hold circuit, a ramp generator and a comparator. The linearity of ATC directly affects the ADC resolution [20]. In this design, OPA is used in the circuit to generate ramp voltage from 1 V to 3 V. Compared with [20], the nonlinearity caused by the finite resistance of current source could be avoided as long as the gain is large enough.

The timing diagram of ATC is shown in Figure 5b. The switches, *S*_1_, *S*_2_ and *S*_3_, are controlled by the *start* signal, which is generated by the timing module. When the *start* signal rises up to high level, *V_ramp_* begins to grow upward from *V*_th_. The switch *S*_4_ is controlled by sample signal, which arrives a little earlier than the *start* signal. When *V_sample_* and *V_ramp_* intersect, the comparator generates a pulse. Assuming that the ramp slope is constant, the time span between the start of the ramp and the comparator pulse is linearly proportional to the input voltage. The output signal of comparator is used as *stop* signal for TDC and the time interval *T*_in_ which will be quantized is between the rising edge of *start* and *stop* signals.

### 3.3. TDC

**Figure 6:**
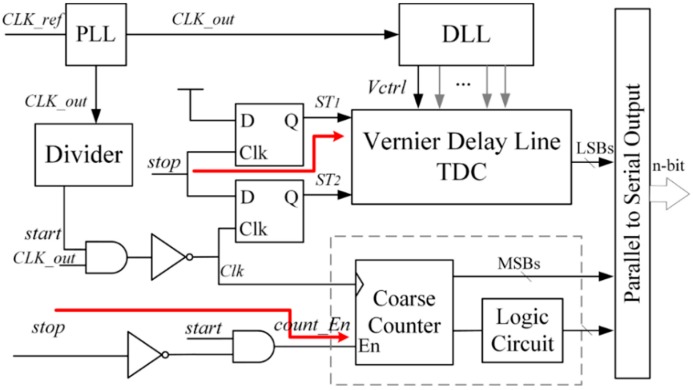
Block diagram of proposed TDC.

**Figure 7:**
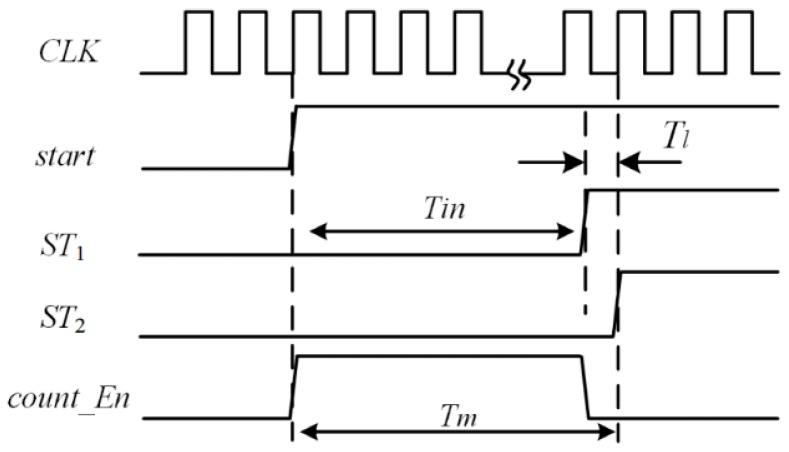
Timing diagram of proposed TDC.

To achieve both energy efficiency and a large dynamic range, a two-step TDC combining a coarse counter and a VDL TDC is proposed. As shown in Figure 6, it is mainly composed of a VDL TDC, a coarse counter, a PLL that stabilizes clock frequency and a DLL to provide control voltages for VDL. A coarse counter quantizes the input time interval coarsely, and the residue is injected into a VDL TDC for fine quantization. Three more signals are introduced to describe the principle well: the start of fine quantization *ST*_1_; the stop of fine quantization *ST*_2_; and the enable signal of coarse quantization *count_En*. *ST*_1_ is set by the rising edge of *stop* signal. *ST*_2_ is set by the next rising edge of clock after the *stop* signal. *count_En* is set by *start* and reset by *stop*. Figure 7 shows the timing diagram of the two-step operation. A coarse counter measures the number of reference clock cycles while the *count_En* signal is high, *i.e.*, it measures *T*_m_. The time interval between the rising edges of *ST*_1_ and *ST*_2_, defined as *T*_l_, is measured by the fine TDC. As shown in Figure 7, the width of the input interval, defined as *T*_in_, is calculated by:

(11) $$T_{\text{in}}=T_{\text{m}}–T_{\text{l}}$$

**Figure 8:**
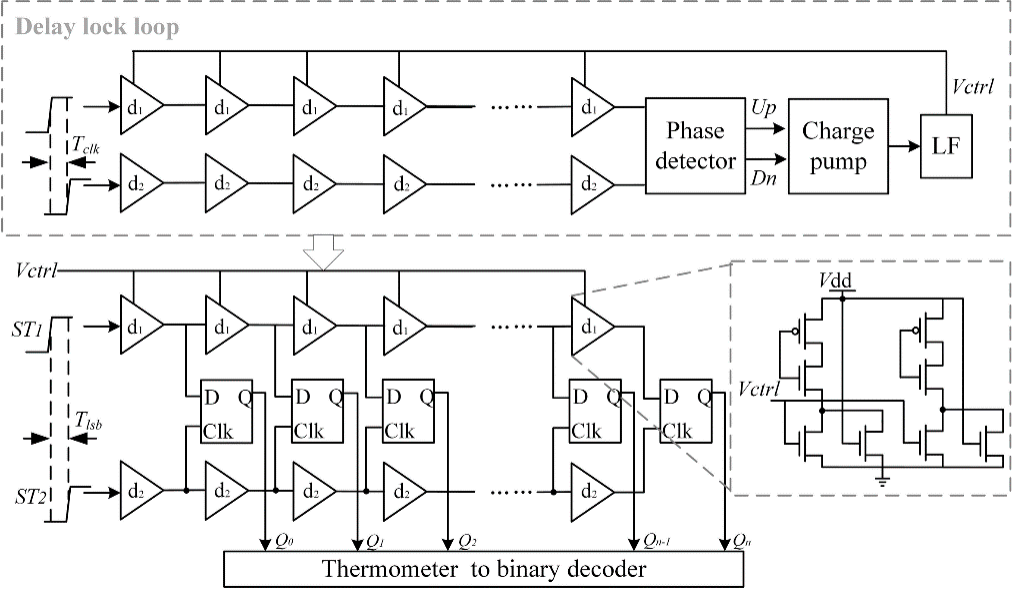
Block diagram of VDL and DLL.

The structure of VDL is shown in Figure 8, it consists of two delay-lines: One for the *ST*_1_ and the other for *ST*_2_. The up-line is composed of voltage-control delay elements and the down-line is composed of fixed delay elements. It is noted that the signal arrival time uncertainty grows with the length of the delay line. If more than 40 stages are implemented in the VDL, manufacture variations are the major sources of errors affecting the linearity. Sixteen stages are chosen for each delay line to quantize *T*_l_.

The delay elements in the up-line have a delay *t*_d1_ which is slightly longer than the delay *t*_d2_ of the elements in the down-line. Thus, when the comparator output *stop* signal arrives, the *ST*_2_ chases the *ST*_1_. In each stage the delay difference is shortened by:

(12) $$△t_{\text{d}}=t_{\text{d}1}-t_{\text{d}2}$$

The order status of two signals is detected by D flip-flops, and the output *Q*_1_*Q*_2_…*Q*_n_ will be “11…00”. Assuming that the amount of “1” is *L*, then the interval of fine quantization is:

(13) Tl = L×△td   = L×(td1−td2)   = L×Tclk2Nl

Finally, this thermometer code will be translated to binary code and *l-*bits digital output is generated.

Thus the TDC output can also be expressed as:

(14) Tin=Tm−Tl    =M×Tclk−L×Tclk2Nl

where *M* is the coarse counter output.

However, the delay difference is uncertain as the digital delay elements depend on process variations, temperature, and supply voltage (PVT variations). The best way to stabilize the value of Δ*t*_d_ against PVT variations and also provide calibration against process variations is to utilize Delay Lock Loop (DLL). DLL has two delay lines just the same as that in TDC as shown in Figure 8. Two similar signals only with one period time offset are injected into the delay lines, respectively. The output signals of the two delay lines are fed to a phase detector (PD) that compares the phase of these periodic signals. The phase detector generates a bias voltage *V*_ctrl_ based on the phase difference to control the delay time, so that the delay of each element can be tuned by its control voltage *V*_ctrl_, and eventually the TDC resolution is locked to: Δ*t*_d_ = *T*_clk_/*N*. A dummy DFF is also implemented in the DLL to balance the loading of the delay elements in the DFF.

### 3.4. Quantization Error and Calibration

**Figure 9:**
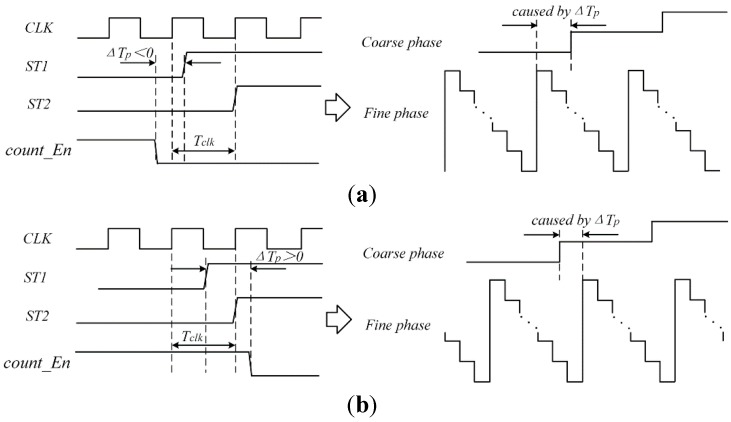
Timing diagrams of the TDC illustrating quantization error sources. (**a**) Δ*T*_p_ < 0; (**b**) Δ*T*_p_ > 0.

For the proposed two-step TDC, the coarse quantization and fine quantization process the input signals in parallel and generate real-time outputs, the synchronization of input signals propagation should be guaranteed. This implies that the falling edge of stop signal of coarse quantization *count_En* must occur in the fine quantization cycle, or the quantization results may have a one-bit coarse quantized error. However, the delay time difference caused by device mismatches and process variations between different propagate paths make it impossible to align the coarse and fine quantization correctly. The quantization errors occur in two conditions, as shown in Figure 9. As depicted in Figure 9a, when the edge of *count_En* is coming before rising edge of the fine quantization clock cycle, the actual result of coarse quantization is 1LSB less than ideal result. On the contrary, when the edge of *count_En* is coming after the fine quantization clock cycle, the actual result of coarse quantization is 1LSB more than ideal result. Therefore, it causes quantization errors and seriously affects linearity.

Some latency can be compensated by additional elements in the propagate path, but additional delay elements also mean additional variability, additional power, and additional noise. In this paper, a calibration method is proposed to eliminate the quantization error with less additional circuits. As shown in Figure 9, the time latency between *count_En* and *ST*_1_, is defined as Δ*T*_p_, and the reasonable range of Δ*T*_p_ is *−T*_clk_~+*T*_clk_.

**Figure 10:**
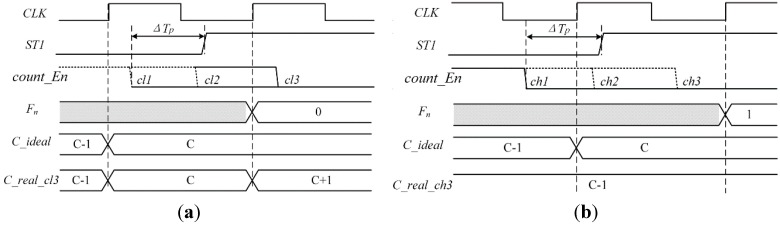
Timing diagram of the proposed calibration circuit. (**a**) *ST*_1_ rises at the low level state of clock; (**b**) *ST*_1_ rises at the high level state of clock (*C_ideal* denotes the ideal output of coarse quantization; *C_real* denotes the actual output of coarse quantization).

As depicted in Figure 10a, when *ST*_1_ rises at the low level state of clock, the edge of *count_En* signal may appear in three positions *cl*_1_,*cl*_2_,*cl*_3_, which indicate that the range of latency is 0~+*T*_clk_, *−T*_clk_/2~+*T*_clk_/2, *−T*_clk_~0, respectively. When *ST*_1_ rises at the high level state of clock, the edge of *count_En* signal may appear in three other positions *ch*_1_,*ch*_2_,*ch*_3_, which indicate that the range of latency is 0~+*T*_clk_, *−T*_clk_/2~+*T*_clk_/2, *−T*_clk_~0, respectively.

**Figure 11:**
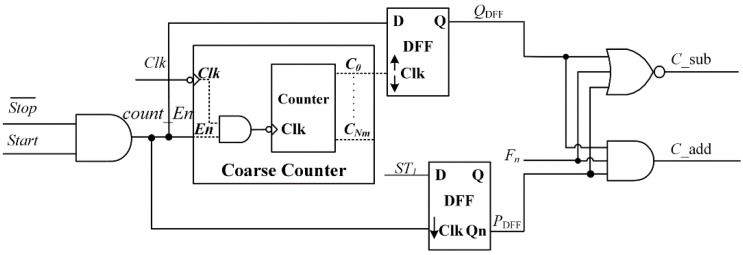
Block diagram of the proposed calibration circuit.

The proposed calibration circuit is illustrated in Figure 11, which is composed of a double input AND, a counter, a double edge-triggered D flip-flop, a falling edge-triggered D flip-flop, a triple input AND and a triple input NOR. The counter is modified as falling edge triggered, thus the last change in the LSB of the counter, *C*_0,_ could be caused by the edge of *count_En* signal. Compensation for coarse quantization result is decided by analyzing logic levels of clock and *ST*_1_, when *stop* signal propagate to the coarse or fine quantization. The double edge-triggered DFF is used to obtain the state of *count_En* signal when *C*_0_ changes. The falling edge-triggered DFF obtains the state of *ST*_1_ when *count_En* falls. Furthermore, the MSB of fine quantitative output, denoted as *F*_n_, is utilized. In the case of *F*_n_ = 0, as shown in Figure 10a, which means *ST*_1_ rises at the low level state of clock. If the falling edge of *count_En* signal appears in position *cl*_3_, which is at the high level state of the clock, the last change of *C*_0_ comes from the falling edge of *count_En*, and the final output of DFF, *Q*_DFF_ = 0. In addition, *ST*_1_ signal is in the high level when *count_En* changes, and it means the reverse output of DFF, *P*_DFF_ = 0. Then the *C*_sub generated by “NOR” operation is high, thus it will subtract 1LSB from the coarse quantization output. Similarly, when the falling edge of *count_En* signal is in position *cl*_1_ or *cl*_2_, the correction bit *C*_sub and *C*_add are equal to 0, which means no need for calibration. On the contrary, ST_1_ rises at the low level state of clock as shown in Figure 10b, in this case, *F*_n_ = 1. If the falling edge of *count_En* signal is in position *ch*_1_, which is in the low level of the clock, the last change of *C*_0_ comes from the falling edge of clock, and the final output of DFF, *Q*_DFF_ = 1. In addition, *ST*_1_ signal is in the low level when *count_En* changes, and it means the reverse output of DFF, *P*_DFF_ = 1. Then the *C*_add generated by “AND” operation is high, thus it will add 1LSB to the output. There is also no need for calibration when the falling edge of *count_En* signal is in position *ch*_2_ or *ch*_3_.

The proposed calibration method can effectively compensate the coarse quantization error caused by delay skew within *−T*_clk_~+*T*_clk_ with a slight increment of area and power consumption.

## 4. Results

**Figure 12:**
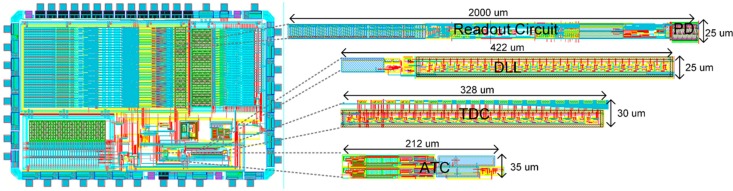
Layout of the linear image sensor.

A linear CMOS image sensor with 32 × 1 pixel array is designed in the 0.13 μm CMOS process. The small-scale array is designed to prove the validity of readout circuits, and the pixel array could be extended, since the proposed readout circuits and ADCs are column wise designs. Figure 12 shows the layout of the linear image sensor. Each column readout circuit consumes 693 μW, and the ADC consumes 355 μW power consumption provided by a 3.3 V supply for the analog circuits and a 1.5 V supply for the digital blocks. The following results are all from post-layout simulation.

### 4.1. Readout Circuits

**Figure 13:**
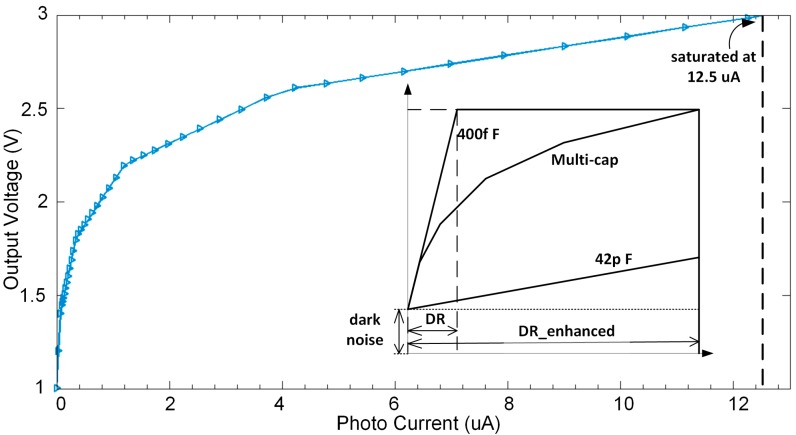
Output voltage *versus* photocurrent. The pixel saturates at around 12.5 μA.

**Figure 14:**
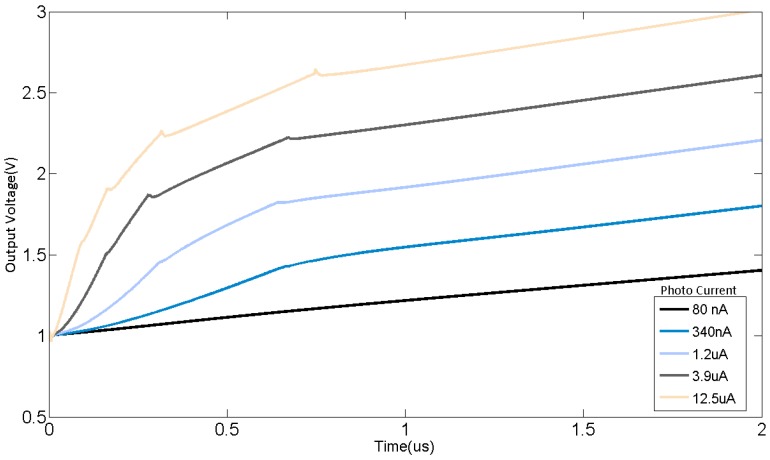
The transient simulation of the pixel integration.

The polyline type photocurrent response curve is shown in Figure 13. As the photocurrent increases, when the output voltage exceeds the threshold voltage each time, the sensitivity gets lower. Finally, the output curve presents as a polyline with five segments as expected. Figure 13 also shows the simulation of traditional CTIA with the same input current, and the integration capacitors are 400 fF and 42 pF, respectively. When the integration capacitor is 400 fF, the CTIA saturates easily and the output signal will no longer keeps linearity with input current, and if the capacitor of 42 pF is accessed to the circuit, the sensitivity is too low to distinguish the low light signals. The transient simulation of the pixel integration readout at different photocurrent is shown in Figure 14. The glitches on the output signal is mainly caused by two reasons: (1) The PD voltage slightly deviates from *V*_PD_RST_ since the OPA is not ideal, which would make a difference between PD voltage and the voltage on the left plate of capacitors; (2) When the OPA outputs large current, the negative terminal of OPA deviates from *V*_ref_, which would also generate offset between PD voltage and the voltage on the left plate of capacitors; (3) The reset noise at the left plate of additional integration capacitors make its voltage various from *V*_PD_RST_, which contributes to the glitches and noise. Noise performance is also simulated, the total noise is 0.7 mV including OPA and KT/C noise induced by capacitors and switches. Then the minimum photo current can be detected is 0.14 nA, and the non-saturated photo current is 12.5 μA. The dynamic range of this readout circuit is extended from 69.11 dB to 99.02 dB.

### 4.2. ADC

**Figure 15:**
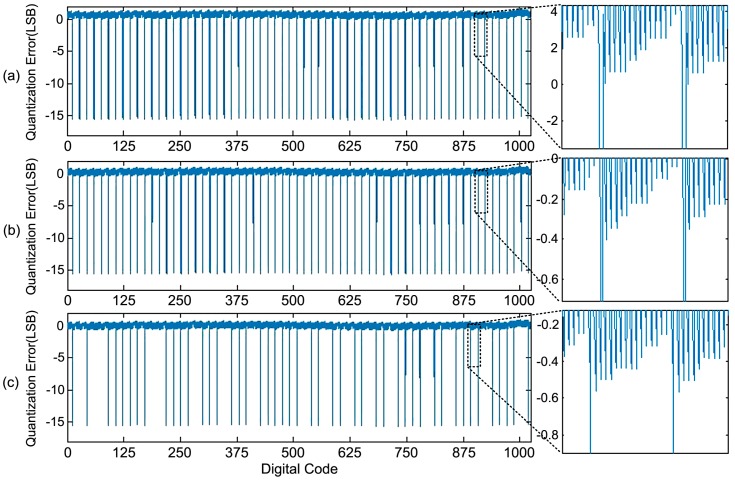
Quantization error without calibration. (**a**) −*T*_clk_ < Δ*T*_p_ < −*T*_clk_/2; (**b**) −*T*_clk_/2 < Δ*T*_p_ < −100 ps; (**c**) −100ps < Δ*T*_p_ < 0.

Different delay skews are simulated by interpolating different dummy loads and delay cells to the propagation path. Simulated quantization error in negative cases (Δ*T*_p_ < 0) without calibration are illustrated in Figure 15.

The quantization error gets better as the delay skew gets shorter, but even in Figure 15c, *i.e.*, when the delay skew is less than 100 ps, the quantization error still exists. The maximum deviation value is +1.78 LSB/−15.76 LSB when −100 ps < Δ*T*_p_ < 0, which the following comparison is based on.

**Figure 16:**
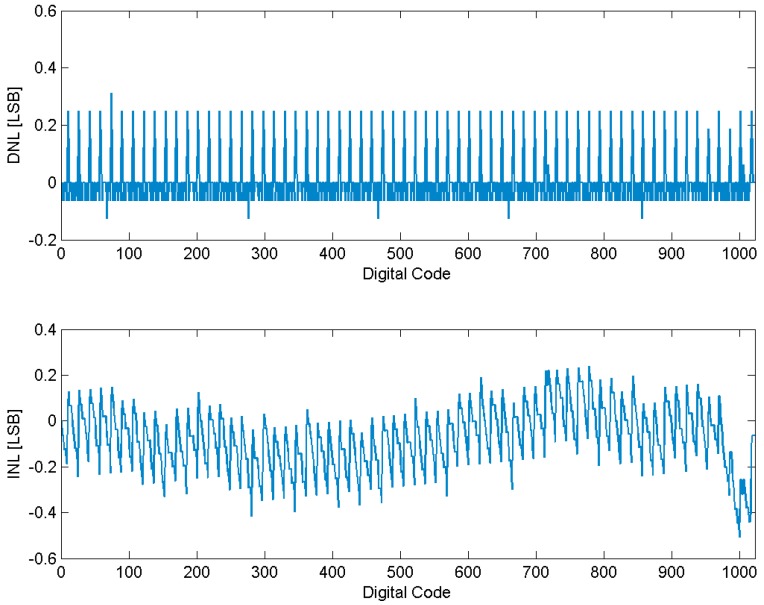
DNL and INL with calibration.

The maximum DNL and INL without calibration is +15.90/−15.36 LSB and +1.78/−15.76 LSB, which is easily obtained from the quantization error result. As shown in Figure 16, the DNL and INL drop to +0.31/−0.11 LSB and +0.25/−0.51 LSB with the proposed error correction.

**Figure 17:**
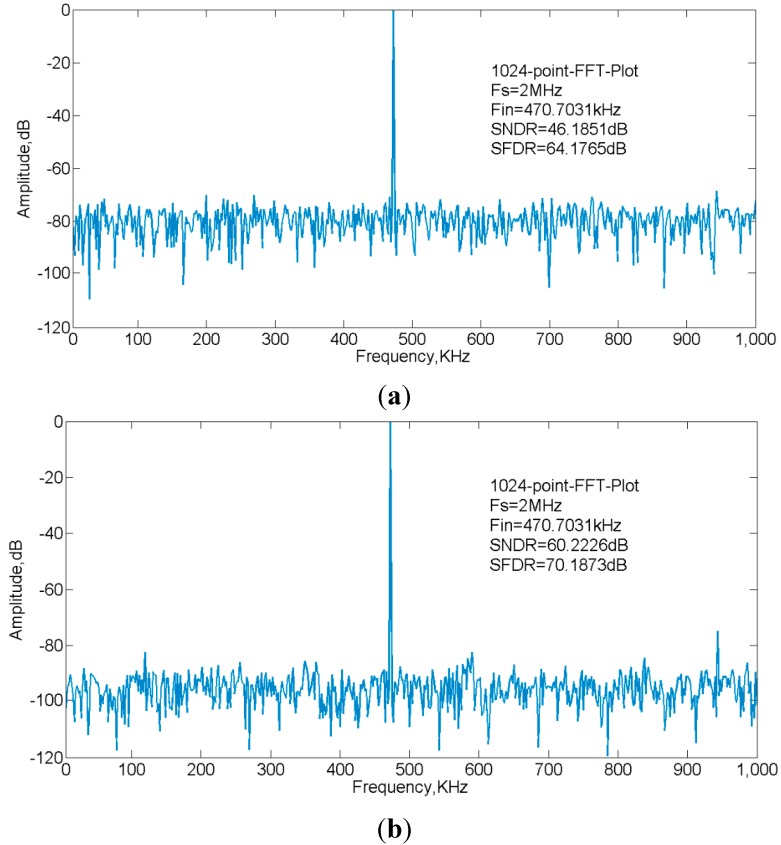
ADC output spectrum. (**a**) Without calibration; (**b**) With calibration.

By applying a 470.7031 KHz sinusoid input sampled at 2 MS/s in the simulation, the 1024-point FFT spectrum shows the ADC achieves 60.22 dB SNDR and 70.18 dB SFDR after calibration while it was limited to 46.18 dB SNDR and 64.17 dB SFDR before calibration, as illustrated in Figure 17. The ENOB measured is 7.37 bit without calibration, which is close to the coarse conversion accuracy. By applying the error calibration, the maximum ENOB has been improved to 9.71 bit. As the sinusoid input frequency decreases, the improvement effect is more remarkable. The comparison of characteristics is summarized in Table 1. The ADC performance has significantly improved with the proposed calibration technique.

**Table 1:** Simulated result comparison.

	Without Calibration	With Calibration
SFDR(@470.7031k), SNDR(@470.7031k)	64.17 dB 46.18 dB	70.18 dB 60.22 dB
ENOB(@470.7031k)	7.31 bit	9.71 bit
SFDR(@1.9531k), SNDR(@1.9531k)	49.87 dB 39.38 dB	69.59 dB 60.19 dB
ENOB(@1.9531k)	6.25 bit	9.72 bit
DNL(max)	+15.90/−15.36 LSB	+0.31/−0.11 LSB
INL(max)	+1.78/−15.76 LSB	+0.25/−0.51 LSB

**Figure 18:**
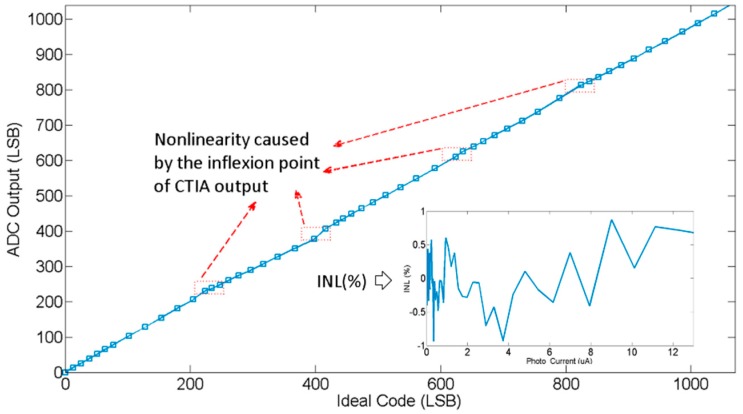
The linearity of the proposed image sensor

The linearity of the proposed image sensor is shown in Figure 18. The ADC output is extracted at different photo current, and the ideal code is calculated by Equation (7) at the same photo current sequence. An overall nonlinearity or INL as a function of photo current is also shown in this figure. The INL includes nonlinearity of the readout circuit and the column-ADC. The maximum INL is +0.87%/−0.91% to the full scale of linear range. As shown in Figure 18, the nonlinearity in marked region is caused by charge injection effect when different capacitors are switched to the circuit. A good linearity could be obtained in every segment if the inflexion point is ignored.

The figure of merit (FOM) is defined as the following equation:

(15) $$FOM_{ADC}=Power/{\text{(2}}^{\text{ENOB}}\times F_{S})$$

where *F_s_* is 2 MS/s, thus the *FOM_ADC_* of each column is 211 fJ/step. Table 2 shows the performance comparisons of various column-parallel ADCs. The proposed ADC works in time-domain instead of voltage-domain, and thus the complexity and power consumption is significantly reduced. Compared with previous works, the proposed ADC achieves high conversion speed and the best *FOM*.

**Table 2:** Comparison with other types of the column-parallel ADC.

Reference	[10]	[12]	[14]	[15]	[18]	This Work
Technology	0.18-μm	0.18-μm	0.18-μm	0.35-μm	0.25-μm	0.13-μm
Architecture	SAR	Cyclic	Single Slope	Two-step SS	Multiple-ramp SS	SS With TDC
Resolution/bit	8	13	12	10	10	10
Conversion time/μs	1.2	6.4	9.5	4	16	0.5
Power/μW	209	450 *	166.7 *	150	91	355
FOM/fJ/step	983.6	442.6	385.9	585.9	1138	211

* Values came from full-chip power consumption.

All the sensor characteristics obtained from simulation results are listed in Table 3. The dynamic range is extended from 69.11 dB to 99.02 dB with nearly 30 dB improvement, and the ADC can effectively operate at 2 MS/s with a calibration method. Therefore, the proposed readout technique can fulfill both high speed and wide range detection features. The maximum line rate is 2 M lines/s if the ADC is implemented as column circuits. Moreover, it can be configured accordingly, e.g., the line rate could be 500 K lines/s if one ADC is shared among four columns.

**Table 3:** Summary of the sensor characteristics.

Technology Photodiode Size	0.13 μm 1P3M 25 μm × 100 μm
Supply Voltage	3.3 V/1.5 V
Line Rate	2 M
Sensitivity	2.5 V/pA·s (max); 0.025 V/pA·s (min)
Noise Floor	0.7 mV
Dynamic Range	99.02 dB
ADC resolution	10 bit
ADC ENOB	9.72 bit (with calibration); 7.31 bit (without calibration)
Power Consumption	1048 μW/column

## 5. Conclusions

A DR enhanced readout technique with two-step TDC for high speed linear CMOS image sensors is proposed in this paper. A multi-capacitor and self-regulated CTIA structure is proposed where the adjustable integration capacitors significantly extend the dynamic range. Moreover, a 10-bit column ADC based on two-step TDC with error calibration is proposed to improve the column conversion rate. The proposed ADC has a significantly increased conversion rate compared with the conventional SS ADC, and requires less power and area than the multiple-ramp ADC. The calibration method can effectively compensate the quantization error caused by the propagation delay skew within −*T*_clk_~+*T*_clk_ without increasing the circuit complexity. Simulation results indicate that the readout circuits can achieve a dynamic range of 99.02 dB with nearly 30 dB improvement, and the ADC can achieve 9.71 bit ENOB at 2 MS/s with 355 μW power consumption. The DNL/INL are significantly reduced down to +0.31/−0.11 LSB and +0.25/−0.51 LSB with calibration. Therefore, the proposed image sensor can fulfill the high speed and wide range detection features.
